# Epidemiological Study on Burnout in Spanish Dentists: Underlying Psychological Factors

**DOI:** 10.3390/ijerph182413418

**Published:** 2021-12-20

**Authors:** Cristina Gómez-Polo, Ana María Martín Casado, Antonio Castaño, Javier Montero

**Affiliations:** 1 Department of Surgery, Faculty of Medicine, University of Salamanca, 37007 Salamanca, Spain; javimont@usal.es; 2 Department of Statistics, Faculty of Medicine, University of Salamanca, 37007 Salamanca, Spain; ammc@usal.es; 3 Department of Stomatology, Faculty of Dentistry, University of Seville, Calle Avicena S/N, 41009 Seville, Spain; acastano@us.es

**Keywords:** burnout syndrome, personality factors, coping styles, dentists

## Abstract

The aim of this work is to study the personality factors and coping styles of Spanish dentists when facing burnout syndrome, through epidemiological work. An epidemiological study of 1928 Spanish dentists was undertaken through an online survey, which registered the age and gender, and three questionnaires: NEO-FFI (personality traits), Brief COPE (coping styles), and MBI-HSS (burnout questionnaire). A multivariate analysis and an explanatory binary logistic regression model were used to estimate the presence of burnout. Neuroticism turned out to be the strongest indicator (OR 1.10; IC 95% 1.09–1.14), while extraversion (OR 0.93; IC 95% 0.91–0.95) and agreeableness (OR 0.94; IC95% 0.92–0.98) showed an inverse relationship with the occurrence of burnout, as did age (OR 0.9; IC95% 0.96–0.99). Conscientiousness (OR 1.0; IC95% 1.00–1.06) had a direct relationship with the presence of burnout. An avoidant coping style was the only indicative coping style (OR 1.04 IC95% 1.02–1.06). Approximately 70% of the dentists scored high on one of the three dimensions of burnout. No differences were found in the degree of burnout according to gender. Certain personality factors (neuroticism, extraversion, agreeableness, and conscientiousness), the avoidant coping style, and age are powerful indicators to attempt to forecast the presence of burnout syndrome in dentists.

## 1. Introduction

“Burnout” or “burnout syndrome” as a concept was described for the first time by the German–American psychiatrist Freudenberger (1974), although Christina Maslach [1] was its greatest disseminator within psychology and the author who has developed the subject the most. Despite the term having been in use for many years, a multitude of definitions of burnout syndrome exist, to the extent of having found 142 different definitions after a recent systematic review; this highlights the lack of consensus [2,3]. A useful definition could be that burnout syndrome represents a complex condition with a multifactorial origin in which the person’s psychological, labor, and relational processes are involved, as well as the mechanisms available to cope with these chronic interpersonal work demands that are perceived as excessive, exceed the resources of the affected individuals, and cause a negative response that reduces wellbeing. This syndrome carries serious consequences for the psychological wellbeing of staff and affects morale and organizational functions at work [4]. In 2000, burnout syndrome was declared by the WHO as an occupational risk factor due to its potential to affect the sufferer’s quality of life, mental health, and even place their life at risk. It is known that professions which require intense contact with other people, such as healthcare workers, teachers, and carers, are more prone to stress and burnout syndrome [5,6,7].

On the other hand, all research indicates that burnout syndrome not only has repercussions on the individual, but will also result in poor service to patients, medical errors, poorer quality care, and higher absenteeism, and will expose the organization to financial losses and lower productivity levels [2,3,8,9,10,11,12].

Healthcare professionals working in the dental sector often face situations of overload [3,7,13] due to emotionally and physically exhausting work. This leads to stress in addition to purely care-related stress [6], where dentists often face conditions of professional loneliness; many also shoulder accounting responsibilities as self-employed workers or sole traders [14]. These last two factors differentiate private dentists from health care staff in hospitals or health centers (who work with other colleagues of the same professional rank and who do not have to complete any financial tasks, as this is handled by hospitals or administration teams). Dental care implies close contact and physical proximity with patients and, in most cases, a relationship of trust, as patients freely choose their dentist. This element also differs from public health care personnel, where the element of “choice” is, in most cases, not contemplated. The effort made by the dentist to gain the trust and loyalty of patients adds an element of stress. It must not be forgotten that one of the main reasons for consultation is orofacial pain, which represents a difficult diagnostic task, as it can involve very diverse anatomical structures and the presence of intense negative emotional alterations. Additionally, aesthetic treatments have increased; these are highly subjective, and the high expectations patients hold of the results can increase the work pressure on dentists. It is worth remembering that dentists work inside the mouth, a small, poorly lit space, which often requires poor posture and a high level of physical proximity to patients. Therefore, the practice of dentistry is considered a stressful profession [15] which can affect the physical and mental wellbeing of dentists and, therefore, the quality and quantity of the treatments they provide [6]. The dental profession, according to many studies in various countries, presents high levels of burnout syndrome [16,17,18]. The percentages differed more in terms of the prevalence of burnout: 3.8% among Spanish dentists [19], ranging from 8 to 36% among dental professionals in European countries [20], 13.6% among periodontists [17], 42% among physicians in the USA [3], and 83.6% among Lithuanian dentists [20]. Research on burnout syndrome has mainly focused on the health sector and not on the dental sector in depth [6,21,22]. Moreover, factors related to work and personal variables, such as age, gender, number of hours worked per week, level of responsibility, years of experience, clinical specialty, etc. have been extensively studied [10,17,21,22,23]; however, the subject’s personal psychological factors, which are potentially modulating, have traditionally been studied the least, and thus relegated to a background position [24]. To identify only the stress produced by work situations as responsible for the appearance of burnout syndrome is a reductionist view, as personal variables which make individuals more vulnerable to burnout syndrome must be considered [24,25,26,27]. Psychological variables and their relationship with burnout have not been previously studied in depth in dentists. In this way, not only has the understanding of burnout processes improved [22,28,29], but also individuals at risk of manifesting symptoms can be identified at an earlier stage [30]. We can define personality as “the predispositions to respond, in the same or similar ways, to different types of stimuli, as congruent and lasting ways of reacting to the environment” [31], in other words, stable/consistent behavioral predispositions, or tendencies to act similarly in certain situations throughout life. This theory is corroborated by various factor studies, most notably by the work of Robert McCrae and Paul Costa in 1990 [32]. Thus, a unified dominant paradigm emerged, known as the “Big Five Personality Model” (FFM for “Five-Factor Model”), determined as the current dominant theoretical underpinning or framework [33], validated and supported by multiple authors [34,35].

Coping with unwanted situations, such as those presented by burnout syndrome, encompasses the cognitive and behavioral efforts necessary to manage and control environmental–external or internal demands that are perceived as negative or stressful to one’s resources [36]. Coping can be defined as the set of cognitive and/or behavioral processes that a person uses to reduce, minimize, tolerate, or control different stressful situations with the aim of managing internal and external or environmental demands in the best possible way. Lazarus and Folkman (1984) state that there are no ideal coping strategies per se, but that the effectiveness and appropriateness of the response should be assessed in terms of their success or failure [36].

Early detection of burnout risk factors would be a central aspect of the assessment, as it allows for the articulation of appropriate intervention and prevention strategies. The present epidemiological study investigates the contribution of personality traits and coping styles in the burnout processes suffered by Spanish dentists, given that there is a lack of studies on the subject [7,27]. As a null hypothesis, we state that there are no psychological factors (personality or coping styles) that affect the prevalence of burnout syndrome in Spanish dentists. The objective was to study the ability to estimate burnout syndrome in Spanish dentists through the analysis of personality factors and coping styles.

## 2. Materials and Methods

All participants were asked to fill in an online survey voluntarily and anonymously through the Dental Associations of all regions of the Spanish national territory, as well as through the General Council of Dentists. Simple sample method was used. The data collection period lasted from 25 May 2019 to 31 December 2019. This study had the approval of the Bioethics Committee of the University (0000356-24.05.2019) and the General Council of Dentists.

In order for the sample to be representative of the population of Spanish dentists, considering the figure by the National Institute of Statistics (2018) of approximately 38,000 Spanish dentists, with a confidence coefficient 95.5%, precision of 3%, and p = q = 0.50, a minimum sample size of 1080 subjects was calculated.

Spanish dentist population (*N* = 37,787):

$$n=\frac{Nz_{\frac{\alpha }{2}}^{2}pq}{d^{2}\left(N-1\right)+z_{\frac{\alpha }{2}}^{2}pq}=\frac{37787\;\times \;2^{2}\;\times \;0.5\;\times \;0.5}{0.03^{2}\;\times \;37786+2^{2}\;\times \;0.5\;\times \;0.5}=1079.4\;\to n=1080$$

To mitigate the risk of not all subjects responding to the survey, oversampling was carried out to ensure a representative sample size as a minimum. A total of 1298 dentists participated in this study, representing a response rate of 3.4%: 517 men (39.8%) and 781 women (60.2%) who completed the three questionnaires and identified their gender and age. The average participant age was 41.9 ± 11.5 years (men 46.4 ± 12.1 years; women 38.9 ± 10.1 years).

The Five-Factor Reduced Personality Inventory (NEO-FFI) by Paul T. Costa and Robert McCrae was used to collect information on personality factors [37]. The NEO-FFI was adapted to the Spanish population by Cordero, Pamos, and Seisdedos (1999) [38]. This tool evaluates five personality factors: neuroticism, extraversion, openness to experience, agreeableness, and conscientiousness. Sixty items make up this reduced version. 1. Neuroticism (N): the counterpoint of which is emotional adjustment and stability; the general tendency is to experience negative feelings, psychological suffering (such as fear, melancholy or sadness, shame, anger, guilt, and disgust). 2. Extraversion (E): extraverted individuals are very sociable, tend to be with people and have a preference for groups and gatherings; they are also assertive, highly capable of enjoyment, active and talkative, and tend to be of a cheerful disposition, and are optimistic, cheerful, and energetic. 3. Openness to experience (O): individuals tend to value experience positively, are interested in the inner world as well as the outer world (exploratory nature); they are intellectually curious, imaginative, and aesthetically sensitive. 4. Agreeableness (A) or cordiality: this factor is very representative of relationships between people (psychosocial bonds, concern for others); it assesses the quality of one’s interpersonal orientation (kind, sensitive, compassionate). 5. Conscientiousness (C): can be considered synonymous with a will to achieve, and individuals who are organized, disciplined, hard-working, responsible, consistent, tenacious, determined, punctual, ambitious, and have goal-directed behavior.

To measure coping styles, the Brief Cope Inventory [39], adapted to the Spanish population by Crespo and Cruzado (1997) [40], was used: this questionnaire comprises 28 items. Likert scales are used to measure how often a certain strategy is applied by the user, with the options being: 1 = never, 2 = occasionally, 3 = nearly always, and 4 = always. This instrument, translated into Spanish, presented a Cronbach’s alpha coefficient of 0.72, both in the Spanish and English versions [41]. These 28 items are grouped into three coping styles according to the factor analysis: 1. Problem-focused coping: aimed at solving the event that is causing stress, characterized by active coping, seeking instrumental support, positive reinterpretation, planning, acceptance, resignation, and humor. 2. Emotion-focused coping: characterized by regulating the emotions associated with the situation that has caused stress, seeking emotional support and discharge. 3. Avoidant coping: characterized by evasive behavior, problem avoidance, self-distraction, religion, denial, substance use, and self-criticism [42,43].

The Maslach Burnout Inventory-Human Services Survey (MBI-HSS), described by Maslach and Jackson in 1996 [44] and adapted by Seisdedos (1997) [45], was chosen as an instrument to measure burnout syndrome, as it was specifically devised for healthcare workers. The scores for each scale were obtained by adding the values of the 22 items. In all of them, the contents and frequency of the feelings described in the items were rated on a 7-point scale (0 = “Never”, 1 = “Few times a year or less”, 2 = “Once a month or less”, 3 = “A few times a month or less”, 4 = “Once a week”, 5 = “Few times a week”, 6 = “Every day”). Burnout syndrome is composed of three dimensions [44]: 1. Emotional exhaustion (EE): characterized by loss of energy, psychological, mental, and physical exhaustion, constant fatigue, and feelings of lack of strength; 2. Depersonalization (DE): characterized by irritability, moodiness, rejection, or negative feelings towards the recipients of the work, emotional detachment, lack of empathy, etc.; 3. Reduced personal accomplishment (RPA): low scores are characterized by low self-esteem, poor work performance, consistently bad character, negative self-perception, sense of failure, avoidance of social relationships, depression, and low ability to solve work and personal problems. Emotional exhaustion (EE) and depersonalization (DE) scores are directly proportional to the intensity of the syndrome. To determine a high level of burnout, the norms established by the manual of the instrument were used [45] with the following scores as MBI cut-off points: over 26 for emotional exhaustion (EE), over 9 for depersonalization (DE), and under 34 in reduced personal accomplishment (RPA). The internal consistency of the questionnaire was estimated with Cronbach’s alpha coefficient for each dimension, resulting in 0.90 for EE, 0.79 for DE, and 0.71 for RPA [46].

Therefore, age, gender, five personality factors (openness to experience, conscientiousness, extraversion, agreeableness, and neuroticism) and three coping styles (avoidant coping, problem-focused coping, and emotion-focused coping) were studied as independent variables. As a dependent variable, the presence of burnout (high scores on at least one of the three dimensions of the MBI-HSS) was considered versus the nonpresence of burnout.

In observational studies, frequencies were used to describe the occurrence of a phenomenon and the probability of an event developing, as well as to measure associations such as the odds ratio (OR) [47]. To compare the mean scores between the two groups of participants (with burnout vs. no burnout) in the independent variables, the independent samples t-test was used, and effect size was assessed and interpreted according to Cohen’s d statistics [48].

When we faced a dichotomous dependent variable (burnout yes/burnout no) that we wanted to estimate, or for which we wanted to assess the association or relationship with other independent and control variables, a logistic regression procedure was completed [49]. For the statistical analysis, the following computer application was used: IBM-SPSS-25 (reference: IBM Corp. Released 2017. IBM SPSS Statistics v 25.0 for Windows; Armonk, NY, USA).

## 3. Results

### 3.1. Prevalence of Burnout, Descriptive Analysis, and Typology of Dentists According to Their Degree of Burnout

**Figure 1:**
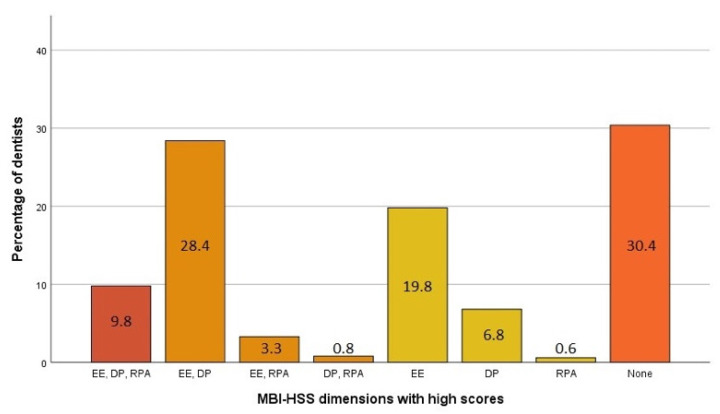
Distribution of dentists participating in our study according to burnout dimensional scores. (MBI-HSS). Emotional exhaustion (EE), depersonalization (DE), and reduced personal accomplishment (RPA).

To analyze the relationship between personality and degree of burnout, considering independently and jointly the three dimensions of the MBI, we classified dentists into four groups (1. Severe burnout: with high scores on all three dimensions of burnout; 2. Moderate burnout: with indications of burnout on two of the three dimensions; 3. Initial burnout: with high scores on one dimension; 4. No burnout: without high scores on any of the three dimensions of burnout) and analyzed the differences in personality between them [50]. For the classification into groups, the normative cut-off points established by the manual of the instrument as outlined above were used [45]. Figure 1 shows the distribution of the participants according to the dimensions of the MBI questionnaire in which they presented high burnout scores.

Thus (Table 1), there were 127 dentists with high burnout scores in all three dimensions (9.8% with severe burnout), 423 dentists with high burnout scores in two dimensions (32.5% with moderate burnout), 353 with high burnout scores in only one dimension (27.2% with initial burnout), and 395 with no high burnout scores in any of the three dimensions (30.4% with no burnout). The largest group was that of dentists with high burnout scores on two of the three dimensions: 369 of the 423 members of this group returned high burnout scores on emotional exhaustion and depersonalization (28.4%). The comparison of the percentages of men and women with severe (10.3 vs. 9.5), moderate (31.5 vs. 33.3), initial (25.9 vs. 28.0), and no burnout (32.3 vs. 29.2) did not show statistical significance (χ^2^ = 1.991, *p* = 0.574). The difference between the percentages of men and women with high burnout scores on at least one of the three dimensions of the MBI-HSS (67.7 vs. 70.8) was not statistically significant either (χ^2^ = 1.420, *p* = 0.233), with an insignificant size effect (OR = 1.16) [51], so gender was not considered an indicator of the syndrome. Table 2 shows the mean, standard deviation, minimum, and maximum dimension scores for the three questionnaires used.

### 3.2. Personality Differences between Groups

We then classified the participating dentists into two groups according to whether or not they obtained high burnout scores: presence of burnout, with high burnout scores on at least one of the three dimensions of the MBI-HSS, 903 dentists (69.5%); and no burnout, without high burnout scores on any of the three dimensions, 395 dentists (30.5%). We compared the groups (burnout/no burnout) in relation to the five dimensions of the NEO-FFI questionnaire. In these analyses, statistically significant differences were obtained between the two groups in the mean scores on all dimensions of the NEO-FFI personality questionnaire, except for openness to experience (Table 3):

- Neuroticism (*p* < 0.001), with a large effect size (d = 1.00) [48]. Mean scores differed from each other, with the burnout group being the highest, with high burnout scores in at least one of the three dimensions.
- Extraversion (*p* < 0.001), with a medium effect size (d = − 0.64) [48]. The highest mean score corresponded to the members of the group without burnout who presented no signs of burnout.
- Agreeableness (*p* < 0.001), with a medium effect size (d = − 0.54) [48]. The highest mean score in this dimension again corresponded to the members of the group without burnout.
- Conscientiousness (*p* < 0.001), with a small effect size (d = − 0.31) [48]. The highest mean score was that of the members of the group without burnout.

### 3.3. Differences in Coping Strategies between the Groups

The comparison of the two groups created on the basis of whether or not they presented high scores on at least one dimension of burnout in relation to the three coping styles derived from the COPE-28 questionnaire indicated that there are statistically significant differences between the two groups in all three coping dimensions (Table 4):

- Problem-focused coping, with a small effect size (d = −0.18) [48]. The mean score was higher for members of the group without burnout.
- Emotion-focused coping, with a small effect size (d = −0.19) [48]. This was the same as in the previous dimension: the highest mean score was that of the members of the group without burnout.
- Avoidant coping (*p* < 0.001), with a medium effect size (d = 0.54) [48]. In this case, the highest mean score was that of the members of the group with burnout.

### 3.4. Correlation Coefficients between the Five Personality Dimensions (NEO FFI), the Three Coping Styles (Brief COPE-28) and Age, and the Three Dimensions of Burnout Syndrome (MBI-HSS)

Table 5 shows the Pearson correlation coefficients between the scores obtained in the five dimensions of the personality questionnaire (NEO FFI) and the three dimensions of the MBI questionnaire. As can be seen, scores on neuroticism correlate positively with EE and DP and negatively with RPA [51]. Notably, avoidant coping scores correlate positively with EE and PD with moderate effect sizes in both cases, and negatively with RPA [51]. RPA correlates positively with problem-focused coping and emotion-focused coping, in both cases with moderate effect sizes [51].

### 3.5. Logistic Regression Model for Estimating Burnout

We then created a logistic regression model to estimate group distribution (burnout group: *n* = 903, with high burnout scores on at least one of the three MBI dimensions; non-burnout group: *n* = 395) from scores on the five dimensions of the NEO-FFI personality questionnaire, the three dimensions of the COPE-28 coping strategies questionnaire, and age. We began by assessing the indicative capacity of each of the independent variables considered individually, and then fitted the multivariate logistic regression model with those that were previously significant. The previous univariate study shows that all personality variables, except openness to experience, and all coping strategy variables are possible indicators of the belonging group (*p* < 0.001). The adjusted multivariate logistic regression model considered globally (Table 6) was statistically significant (χ2 = 386.899, *p* < 0.001) and had a Nagelkerke’s R2 coefficient of 0.364. The overall percentage correctly classified was 76.7%, classifying dentists with burnout (88.2%) better than dentists without burnout (50.6%). Finally, the statistical value of the Hosmer–Lemeshow goodness-of-fit test did not reach statistical significance (*p* = 0.821), so the null hypothesis that the fit is good could not be rejected.

In the adjusted model, gender and the dimensions problem-focused coping and emotion-focused coping were excluded because they were not statistically significant in the multivariate model.

## 4. Discussion

Although existing research on the origin of burnout has paid special attention to organizational factors, it should be noted that personal variables not only exert an important influence on the appearance of burnout [30], but also on the predisposition and modulation of the subject to the syndrome. The null hypothesis must be rejected because age, personality factors (except openness to experience), and an avoidant coping style were significant indicators of burnout syndrome in dentists. Therefore, it is unavoidable to consider the interaction that is established between the elements of the work environment and the aspects related to the psychological variables themselves. For Cordes et al. (1993) [52] and for Swider et al. (2010), [53] the exclusion of personal aspects is an indicator of an insufficient approach and, as the present results showed, they should not be excluded as they hold important power. It is important to highlight the importance of carrying out future multicausal analyses, where a greater number of variables modulating burnout, such as personal variables (marital status, number of children, etc.), other psychological variables (engagement, level of motivation, etc.), work variables (number of working hours, own or external consultation, etc.), and sociocultural variables (individualistic or collectivist society) are collected for a better understanding of burnout, to thus be able to identify and prevent it more efficiently. The aforementioned variables have not been considered in this study.

It is unknown how the syndrome evolves over time, as there have been few published longitudinal studies [54,55,56], and it would be desirable to design research with these characteristics [11]. The present epidemiological study may serve as a reference for the state of Spanish dentists before COVID-19. It is worrying that 7 out of 10 dentists presented an affected burnout dimension; this entails a great risk when facing a pandemic situation, which is likely to aggravate the syndrome.

The dimension with the most affected dentists was emotional exhaustion (EE) with almost 20%, while the dimension with the lowest representation was reduced personal accomplishment (RPA) with 0.6%. The results published about dentists by Molina et al. [19] and Rios-Santos et al. [17] were concordant: the dimension most affected by dentists was emotional exhaustion (EE) with 45.6% and 40% respectively, and the least affected was reduced personal accomplishment (RPA) with 8.5% and 11.2% respectively. The percentages were similar for nurses: 23% reported high levels of emotional exhaustion (EE), 11.4% reported high levels of depersonalization, and 12.8% reported reduced personal accomplishment [54]. Once the percentages of affectation by dimensions were analyzed, it seemed that the dimension of burnout least affected in dentists was reduced personal accomplishment (RPA) [19], although differences were observed in the figures, with the present results showing a low 0.6% vs. 8.5% in Molina et al. [19]. It would be desirable for the research to consider the different degrees of burnout in order to determine more precisely the state of affectation and severity of the syndrome among professionals.

It would be advisable to investigate more closely the age ranges in which the incidence of burnout was found, as work productivity is expected to peak between the ages of 35 and 55. The relationship between burnout and age has been studied in the past, and although data was not conclusive, a high percentage of studies [11,28,57,58,59,60,61] defended the inverse connection between age and burnout, indicating that subjects experience lower levels of burnout as their age increases, in agreement with the results of this research, where age was a significant indicator of burnout. This inverse relationship between greater professional experience and lower levels of burnout could possibly be attributed to greater resilience among middle-aged dentists, as past experiences allow them to work better under stress and develop more tailored coping skills [62] or to older workers’ commitment to work. [16] In this line, Schaufeli and Enzmann (1998) [63] emphasize that the development of burnout symptoms in young employees is due to circumstances such as reality shock, high expectations, or low success when socializing. However, Bui et al. (2011) [64] conclude in their study that no relationship between age and burnout is identified. In contrast, the demographic variable gender is not proposed as a strong indicator of burnout [59,65], as supported by our results. The sample sizes of men and women were similar in this study, as there was a greater number of women; this greater presence of the female gender in the dental profession was a true reflection of the higher number of female dentists than male dentists registered in Spain, as the demographic profile has gradually changed [16]. In this sense, some authors suggested that the data does not demonstrate the impact of gender on the dimensions of burnout [61,66]. On the contrary, other analyses [11,28,57,59,66] indicated that emotional exhaustion tended to be more common among women, while depersonalization was present to a greater extent among men [45]. Coincidentally, it was also men who experienced a higher degree of personal accomplishment [67]. The reasons for these differences could be a consequence of established gender role stereotypes and different perceptions of the work environment [28,68]. The results of this study showed that men were more likely to experience a higher degree of personal accomplishment than women.

Dentists with signs of burnout in at least one of the three dimensions, emotional exhaustion, depersonalization, and reduced personal accomplishment, presented higher scores on neuroticism and lower scores on extraversion, agreeableness, and conscientiousness than dentists without signs of burnout. Scores on openness to experience did not differ between groups with and without burnout as published by Piedmont et al. in 1993 [69]. The vast majority of publications were consistent in highlighting neuroticism (low emotional stability) as the main personality factor directly related to burnout syndrome [54,55,56,70,71,72]; in addition, as neuroticism increased, emotional exhaustion and depersonalization also increased in studies on nurses [54,70]. In contrast, extraverted people manifested positive emotions, the need to express themselves, and a high frequency and intensity of personal interactions [30]. Consequently, these considerations seemed to indicate that high levels of extraversion are associated with the perception of a more favorable environment. More specifically, findings revealed that there is a negative relationship between extraversion and burnout dimensions [29,69], as reflected in our results. However, not only did extraversion act as a protective personality factor, but so did agreeableness [56,64] and conscientiousness [29,30,64,69]. The literature does not provide a determinant relationship between personality factors and burnout syndrome [30]. In order to approach this knowledge, there is an insistence on incorporating these individual psychological factors into research as possible triggers of burnout [29,73]. Based on the complex nature of the syndrome [43], the temporal relationship between the three dimensions of burnout has not been defined by the scientific community. Although emotional exhaustion can be considered the first symptom of the burnout process [45], depersonalization is considered a maladaptive coping strategy [74], which increases the progression of burnout [75]; reduced personal accomplishment could be a consequence of emotional exhaustion [76]. Coping processes, in general terms, start with the feeling of threat or loss, which is coped with by investing in resources [77,78]. If the investment is not successful, individuals begin to manifest stress-related aspects [78] and an increasing spiral can be entered that can lead to the development of burnout [77,79], which introduces the approach of linking the burnout process with coping behaviors. Taking into account that burnout could be a response to stress resulting from poor management of coping strategies, we found published results that supported the link between emotion-focused coping and emotional exhaustion and between emotional coping and reduced personal accomplishment, [54,80,81] but not the present results. On the other hand, we can support the relationship between avoidant coping strategies and burnout syndrome, as avoidance itself is a negatively reinforcing behavior [82,83]. This is consistent with the results presented, as dentists with high scores on at least one of the three burnout dimensions presented lower scores on problem-focused coping and emotion-focused coping and higher scores on avoidant coping than dentists without signs of burnout. Van de Broeck et al. (2010) [84] argue that a problem-focused coping style favors the achievement of work goals.

Considering the strength of the association of the independent variables studied with burnout syndrome, we can affirm that, according to our results, the element with the strongest association was neuroticism and the one with the weakest association was age. The association is positive or direct in the case of neuroticism, avoidant coping, and conscientiousness, and negative or inverse in the case of extraversion, agreeableness and conscientiousness and age. The strongest indicator of the presence of any of the burnout dimensions was neuroticism, followed by extraversion with half as much power and inverse effect. Avoidant coping and agreeableness had practically the same weight in the presence of burnout, acting directly and inversely, respectively. It should be noted that there are other variables not studied in this manuscript, mentioned above, which may modify the effect ranges described in this research. Despite the great usefulness of the logistic regression analysis to estimate yes/no for the presence of a syndrome, it had been rarely applied to burnout. One of the few studies with this statistical method applied to dentists is the one published by Yansane et al., but we cannot compare the results directly because it only studies work variables in their relationship with errors in clinical practice [85]. The number of participants in the present study (*N* = 1298) was higher than in other similar studies in the dental field (*N* = 366 dentists [19], *N* = 231 [14], *N* = 492 [16], *N* = 284 [47], *N* = 35 [86]); moreover, the present sample was representative of the total population of Spanish dentists both in number of subjects and geographical distribution of the Spanish territory, as all the provinces were represented.

This cross-sectional study did not include organizational factors at work, as these have been extensively studied as an influence on the occurrence of burnout syndrome [3,8,9,10,11,12]; however, it focused on individual factors linked to the specific traits of those most vulnerable to experiencing burnout symptoms [28]. Companies or self-employed workers can choose to interfere with and stop burnout triggers, and/or, on the other hand, provide coping strategies to employees who are more vulnerable due to their individual psychological characteristics. Burnout interventions can focus on the individual and try to increase employees’ psychological resources, improve coping with stressors at work, reduce unfavorable work factors, try to change the work context, and reduce the sources of stress [63].

Burnout has adverse work-related physical and mental health effects, but there is no consensus on how to treat it [58]. Therefore, there are numerous types of treatment that contribute to reducing the distress caused by burnout: cognitive coping training or a social support group (90 min per week for seven weeks) [87], mindfulness [54], training in basic and advanced problem-solving skills and cognitive restructuring procedures in the face of stress, positive thinking and boundaries [88], consideration of work–life boundaries [89], and interventions in coping strategies [90].

## 5. Conclusions

Considering the limitations of this study, we can conclude that neuroticism and avoidant coping behaviors are indicators of an increased vulnerability to burnout. There are also protective factors, such as extraversion, agreeableness, conscientiousness, and age (the older the employee, the lower the presence of burnout).

## Figures and Tables

**Table 1:** Distribution of the dentists participating in the study according to their degree of burnout severity.

Prevalence	Number of Dentists	Percentage (%)
No Burnout	395	30.5%
Initial Burnout	353	27.2%%
Moderate Burnout	423	32.5%
Severe Burnout	127	9.8%

**Table 2:** Descriptive statistics for age and the scores obtained in the Five-Factor Reduced Personality Inventory (NEO FFI), the Brief Cope Inventory (COPE-28), and the Malasch Burnout Inventory-Human Services Survey (MBI-HSS).

	Mean	SD	Minimum	Maximum
Age	41.8	11.5	22	74
NEO FFI				
Neuroticism	33.8	9.2	12	60
Extraversion	40.2	7.3	15	60
Openness to experience	40.1	6.8	20	60
Agreeableness	42.1	5.9	15	60
Conscientiousness	45.9	6.0	27	60
COPE-28				
Problem-focused coping	70.9	10.4	25.0	100.0
Emotion-focused coping	56.2	8.9	25.0	87.5
Avoidant coping	48.8	7.5	25.0	77.1
MBI-HSS				
Emotional exhaustion	30.8	10.9	9	54
Depersonalization	10.3	4.7	5	28
Reduced personal accomplishment	39.8	5.9	8	48

**Table 3:** Means and standard deviations, independent samples t and *p* values, and magnitude of the difference between groups between groups in the dimensions of the NEO-FFI personality questionnaire, classified by whether high scores in at least one dimension of burnout or no burnout were detected.

Dimension	Burnout (*n* = 903) Mean (SD)	No Burnout (*n* = 395) Mean (SD)	T Test	*p*	Cohen’s d (CI95%)
Neuroticism	36.6 (8.6)	27.5 (7.0)	18.652	<0.001	1.00 (0.89; 1.10)
Extraversion	38.8 (7.4)	43.5 (6.1)	−11.128	<0.001	−0.64 (−0.75; −0.53)
Openness to experience	39.9 (7.0)	40.4 (6.3)	−1.220	0.223	−0.07 (−0.19; 0.04)
Agreeableness	41.1 (5.9)	44.3 (5.2)	−9.328	<0.001	−0.54 (−0.66; −0.43)
Conscientiousness	45.3 (6.1)	47.2 (5.8)	−5.242	<0.001	−0.31 (−0.43; −0.20)

**Table 4:** Means and standard deviations, independent samples t and *p* values, and magnitude of the difference between groups in the dimensions of the COPE-28 coping questionnaire, classified by whether high scores in at least one dimension of burnout or no burnout were detected.

Dimension	Burnout (*n* = 903) Mean (SD	No Burnout (*n* = 395) Mean (SD)	T Test	*p*	Cohen’s d (CI95%)
Problem-focused coping	70.4 (10.7)	72.2 (9.7)	8.733	0.003	−0.18 (−0.30; −0.06)
Emotion-focused coping	55.7 (9.0)	57.4 (8.6)	9.826	0.002	−0.19 (−0.31; −0.07)
Avoidant coping	50.0 (7.7)	45.9 (6.3)	87.034	<0.001	0.54 (0.43; 0.66)

**Table 5:** Pearson correlation coefficients between the scores in each of the three dimensions of MBI-HSS and the remaining variables analyzed.

Pearson’s Correlation Coefficients			
	Emotional Exhaustion	Depersonalization	Reduced Personal Accomplishment
Age	−0.140 **	−0.131 **	0.080 **
NEO FFI			
Neuroticism	0.633 **	0.381 **	−0.508 **
Extraversion	−0.395 **	−0.309 **	0.471 **
Openness to experience	−0.038	−0.043	0.190 **
Agreeableness	−0.267 **	−0.355 **	0.267 **
Conscientiousness	−0.189 **	−0.144 **	0.322 **
COPE-28			
Problem-focused coping	−0.086 **	−0.060 *	0.309 **
Emotion-focused coping	−0.085 **	−0.053	0.281 **
Avoidant coping	0.340 **	0.327 **	−0.146 **

* 0.01 < *p* < 0.005/** *p* < 0.01.

**Table 6:** Logistic regression model adjusted to estimate whether burnout was present or not. Indicators: personality variables except openness to experience (NEO-FFI), coping strategy variables (COPE-28), and age.

	B	DE(B)	Wald	*p*	OR	CI 95% OR
Neuroticism	0.115	0.011	102.793	<0.001	1.122	1.098–1.148
Extraversion	−0.066	0.012	28.268	<0.001	0.936	0.914–0.959
Agreeableness	−0.047	0.014	11.744	0.001	0.954	0.928–0.980
Avoidant coping	0.043	0.011	14.808	<0.001	1.044	1.022–1.068
Conscientiousness	0.035	0.013	6.580	0.010	1.035	1.008–1.063
Age	−0.020	0.006	10.026	0.002	0.980	0.968–0.992
